# Myeloid dendritic cells correlate with clinical response whereas plasmacytoid dendritic cells impact autoantibody development in rheumatoid arthritis patients treated with infliximab

**DOI:** 10.1186/ar2746

**Published:** 2009-06-29

**Authors:** Christophe Richez, Thierry Schaeverbeke, Chantal Dumoulin, Joël Dehais, Jean-François Moreau, Patrick Blanco

**Affiliations:** 1 Département de Rhumatologie, CHU Bordeaux, place Amélie Raba-Léon, 33076 Bordeaux, France; 2 UMR-CNRS 5164, Université Bordeaux 2, 146 rue Léo Saignat, 33076 Bordeaux, France; 3 Département de Virologie et d'Immunologie biologique, CHU Bordeaux, place Amélie Raba-Léon, 33076 Bordeaux, France; 4 Service de Médecine Interne, CHU Bordeaux, 1 Avenue de Magellan, 33600 Pessac, France

## Abstract

**Introduction:**

The objective of our study was to identify the significance of the subtypes of dendritic cell (DC), specifically myeloid DCs (mDCs) and plasmacytoid DCs (pDCs), in rheumatoid arthritis (RA) pathogenesis through their longitudinal follow-up in patients receiving infliximab.

**Methods:**

Circulating mDC and pDC levels were evaluated by flow cytometry in RA patients (n = 61) and healthy volunteers (n = 30). In RA patients, these levels were measured before and during infliximab therapy. Their counts were correlated to RA disease activity markers and anti-nuclear antibody occurrence. IFNα production was measured by ELISA in serum of RA patients and, *in vitro*, in supernatant of peripheral blood mononuclear cells stimulated by influenza virus in the presence or absence of infliximab. Statistical evaluations were based on Mann–Whitney tests or Wilcoxon's signed-rank tests.

**Results:**

RA patients with active disease were characterized by a baseline decrease in both circulating pDCs and mDCs. Disease activity markers inversely correlated only with mDC level. This level increased in RA patients responsive to infliximab therapy, to reach the level observed in controls. Conversely, anti-nuclear antibody appearance during infliximab therapy correlated inversely with pDC level and was associated with increased serum IFNα level and circulating plasma cells number. *In vitro *studies revealed that infliximab kept pDCs in an IFNα secreting state upon viral stimulation allowing differentiation of B cells into anti-nuclear antibody-secreting plasma cells.

**Conclusions:**

This study reveals two distinct roles for pDC and mDC in RA. Circulating mDCs mainly contribute to RA activity, whereas pDCs seem to be involved in appearance of anti-nuclear antibodies under infliximab therapy through the ability of this drug to keep pDCs in an IFNα secreting state.

## Introduction

Dendritic cells (DCs) represent a critical link between innate and adaptive immune systems. Two DC subsets, myeloid dendritic cells (mDCs) and plasmacytoid dendritic cells (pDCs), have been identified in humans. These DC subsets recognize different microbial pathogens through specific receptors, which in turn induce different types of innate and adaptive immune responses [1]. Abnormalities of DC homeostasis have been involved in the pathophysiology of various human diseases, including autoimmune diseases [2]. In systemic lupus erythematosus (SLE), an autoimmune disease characterized by the presence of an autoimmune reaction against nuclear components, pDCs secrete large amounts of IFNα. This secretion promotes the differentiation of monocytes into mDCs. These mDCs capture circulating nucleic acid-containing bodies and activate autoreactive T cells and B cells, leading to the increased production of autoantibodies by plasma cells [3,4].

Rheumatoid arthritis (RA) is a common inflammatory disease, yet its pathogenesis remains incompletely understood. It is probable that DCs could play a key role in its pathogenesis as they have been reported to infiltrate the synovium in RA patients [5,6]. These synovial DCs are more mature than DCs from peripheral blood: they express various activation markers, secrete large amounts of various cytokines (IL-12, TNFα, IL-6), and are able to activate autologous T lymphocytes as well as B lymphocytes [7-9]. Trying to dissect and decipher the exact roles of mDC and pDC subsets in this disease, however, remains difficult because both subsets are present in RA synovial fluid and infiltrate synovial tissues [10,11].

Anti-TNFα therapies have improved the prognosis of RA, although these agents may induce a number of adverse effects including autoimmunity. Anti-nuclear antibodies (ANAs) develop in 30 to 60% of the patients given anti-TNFα regimens [12-14] and, occasionally, clinical lupus develops during the course of therapy [15,16]. The mechanism responsible is still unclear. The TNF/TNF-receptor system appears to play an important role in SLE pathogenesis, as is exemplified by TNFα-induced amelioration of murine lupus nephritis [17] and an increased soluble TNF-receptor correlation with disease activity [18]. These data suggest a role of anti-TNFα in exacerbation or induction of lupus-type autoimmunity and, therefore, could explain some events occurring in patients treated by TNFα blockers. Despite these observations, a recent study has suggested that SLE can be treated with infliximab, although autoantibodies to double-stranded DNA and cardiolipin were increased [19].

To understand the implication of DC subsets in RA immunopathology, we examined peripheral pDC and mDC numbers in patients suffering from active RA and the evolution of these numbers during the course of infliximab treatment. Our study demonstrates that RA activity correlates with fluctuations in mDC numbers and reveals a possible role for the pDCs, through their sustained IFNα production, in the ANA production induced by infliximab.

## Materials and methods

### Study population

Sixty-one patients with active RA (Disease Activity Score in 28 joints (DAS28) >5.1), who fulfilled the revised classification criteria of the American College of Rheumatology for RA [20], were evaluated before and after infliximab therapy. Table 1 summarizes the characteristics of these patients.

**Table 1:** Baseline characteristics of the study patients

Characteristic	Baseline value
Age (years)	55 (24 to 82)
Sex ratio (female:male)	7:3
Disease status	
Disease duration (years)	14 (2 to 34)
Rheumatoid factor positivity (%)	76
Anti-cyclic citrullinated peptide positivity (%)	59
Disease Activity Score in 28 joints score	6.14 ± 1.38
C-reactive protein (mg/l)	36.2 ± 30.5

Data presented as mean (range) or mean ± standard deviation unless otherwise indicated.

Infliximab (Shering-Plough, Levallois-Perret, France) was given at a dose of 3 mg/kg intravenously at weeks 0, 2 and 6 and then every 8 weeks in combination with stable doses of methotrexate 7.5 to 15 mg/week orally or intramuscularly. Only patients on stable prednisone doses ≤ 10 mg/day and nonsteroidal anti-inflammatory drug treatment were included. According to EULAR response criteria [21], a positive clinical response to infliximab therapy was defined as a drop in the DAS28 from baseline by >1.2 or as a DAS28 <3.2 at week 14.

In addition, 30 healthy blood donors were included in the study. These donors were matched with patients for sex and age. Synovial fluid was obtained from 11 patients suffering from osteoarthritis.

The study was approved by the local Ethics Committee, and all patients gave informed consent.

### Enumeration of blood dendritic cell precursors and plasma cells by flow cytometry

Whole blood samples were analyzed on a FACSCalibur flow cytometer (BD Biosciences, Pont-de-Claix, France) with 10^6 ^white blood cells acquired per analysis. DC subsets were measured using a DC kit from BD Biosciences. Peripheral blood mDC and pDC subsets were defined by the concomitant lack of lineage markers, HLA-DR expression, and mutually exclusive membrane expression of CD11c or CD123, respectively. Absolute numbers of blood DC precursors were calculated as the percentage of white blood cells expressed per milliliter of peripheral blood. Enumeration of blood DC was evaluated as published elsewhere [22]. Plasma cells were analyzed by gating on CD19^+ ^cells and by calculating the percentage of CD20^neg^/CD38^high ^cells.

Synovial fluid was obtained at the initial time point from patients with RA (n = 9) and from patients with osteoarthritis (n = 11), with knee effusions. This synovial fluid was diluted appropriately with PBS in order to avoid clot formation. Synovial mDC and pDC subsets were defined by the concomitant lack of lineage markers (CD3^-^, CD14^-^, CD16^-^, CD56^-^, CD8^- ^and CD19^-^), HLA-DR expression, and mutually exclusive membrane expression of CD11c or CD123, respectively. Results were expressed as the percentage of mDCs or pDCs among cells without the following lineage markers: CD3, CD14, CD16, CD56, CD8 and CD19.

### IFNα quantification

Serum samples were collected and were stored at -80°C. IFNα levels were quantified with a human IFNα ELISA kit (BioSource International, Camarillo, CA, USA), according to the manufacturer's instructions. The detection limit of this IFNα ELISA is 25 pg/ml. This assay has been used previously by others groups for measurement of IFNα in the serum [3,23].

### Preparation of cell culture

Peripheral blood mononuclear cells (PBMCs) of adult donors were isolated using Ficoll-Paque Plus (Amersham Biosciences, Saclay, France) gradient centrifugation. PBMCs (1 × 10^6 ^cells/well) were cultured in RPMI supplemented with 10% FCS, and were stimulated *in vitro *with live influenza virus (10^4 ^particles; Charles River Laboratories, Wilmington, MA, USA) with or without TNFα (10 μg/ml; R&D Systems, Lille, France) or TNFα blockers (Infliximab 20 μg/ml; Shering-Plough) in 96-well U-bottom plates. The infliximab dose used *in vitro *is comparable with the infliximab serum concentration found *in vivo *during the first weeks after the infusion [24]. After 24 hours incubation, supernatants were collected. Depending on the conditions, cells were further incubated in fresh RPMI with live influenza virus (10^4 ^particles; Charles River Laboratories). After 24 hours, the supernatants were again collected for IFNα quantification by ELISA.

### Plasma cell generation and antibody production

PBMCs were isolated by Ficoll-Paque Plus (Amersham Biosciences, Saclay, France) gradient centrifugation – from RA patients treated by infliximab who had developed significant ANA titers, from healthy donors and from SLE patients. PBMCs (1 × 10^6^/well) were then cultured with 10^4 ^influenza virus particles (Charles Rivers, Wilmington, MA, USA) with or without TNFα (10 μg/ml; R&D Systems) or TNFα blockers (Infliximab 20 μg/ml; Schering-Plough, Levallois-Perret, France) in a 48-well plate in 10% FCS RPMI supplemented with rhIL-2 (50 U/ml; R&D Systems, Lille, France). At day 15, supernatants were collected and tested for ANAs. The resulting B cells were analyzed using flow cytometry after gating on CD19^+ ^cells and by calculating the percentage of CD20^low^/CD38^high ^cells.

### Statistical analysis

Statistical analysis was performed using the GraphPad InStat software (version 3.0a for Macintosh; GraphPad Software, San Diego, CA, USA). Mann–Whitney tests were used for mean comparisons between groups. Wilcoxon's signed-rank test was used for the analyses of matched pairs. Correlation between DCs and activity markers were assessed using linear regression, given with the *r*^2 ^correlation coefficient. *P *< 0.05 was considered statistically significant.

## Results

### Blood dendritic cell subsets in RA and their correlation with disease activity

**Figure 1:**
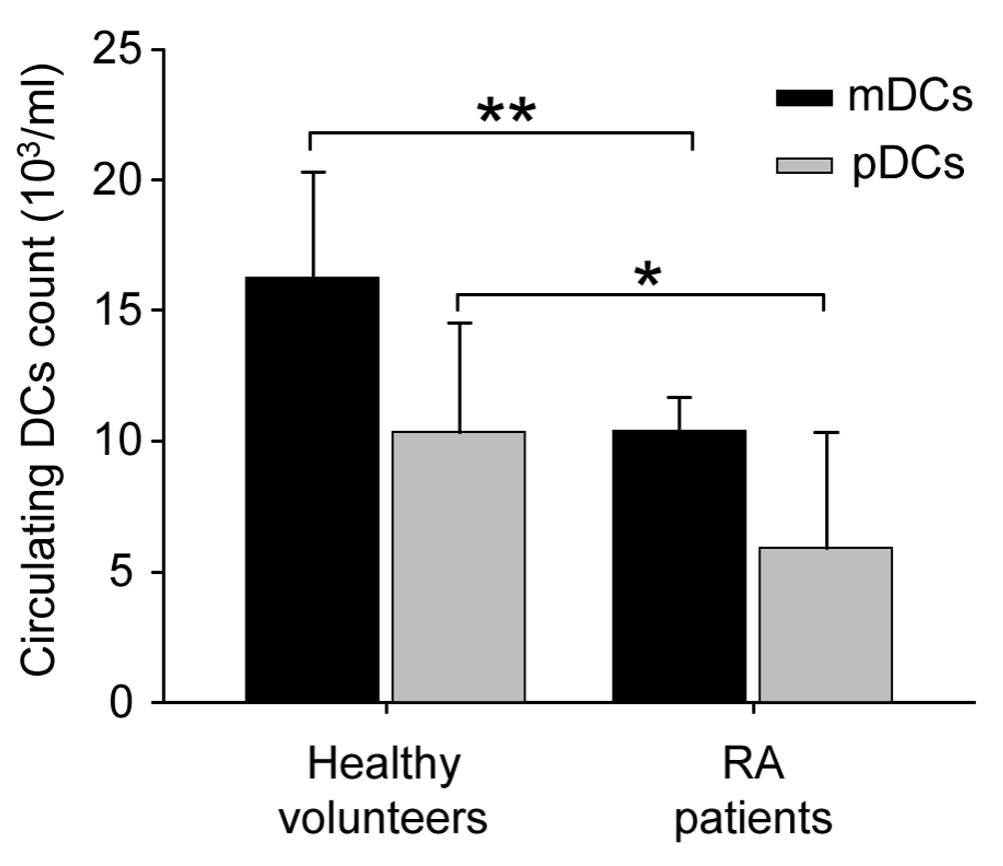
Circulating dendritic cell subset levels in patients with active rheumatoid arthritis and in healthy volunteers. Dendritic cell (DC) subsets were measured in the peripheral blood of patients with rheumatoid arthritis (RA) (n = 61) and in healthy subjects (n = 30). The mean numbers per milliliter of blood of CD11c^+^CD123^-^Lin^-^HLA-DR^+ ^myeloid dendritic cells (mDCs) and CD11c^-^CD123^+^Lin^-^HLA-DR^+ ^plasmacytoid dendritic cells (pDCs) are shown (mean ± standard deviation). *P < 0.0001 and **P < 0.001, Mann–Whitney U test.

To better delineate the involvement of known DC subsets in RA pathogenesis, we compared the number of circulating CD11c^+^HLA-DR^+^CD123^- ^mDCs and CD11c^-^HLA-DR^+^CD123^+ ^pDCs in peripheral blood from 61 active RA patients (free of TNFα-blocker treatment) and from 30 healthy volunteers. Interestingly, RA peripheral blood was characterized by a decreased number of both pDC and mDC subsets (mean ± standard deviation): mDC count = 10,214 ± 7,576 cells/ml in the RA group versus 16,228 ± 4,057 cells/ml in the healthy control group (*P *= 0.0002), and pDC count = 6,098 ± 4,710 cells/ml in the RA group versus 10,313 ± 4,201 cells/ml in the healthy control group (*P *< 0.0001) (Figure 1). We concluded that RA patients are characterized by a quantitative deficit in their peripheral circulating DCs.

**Figure 2:**
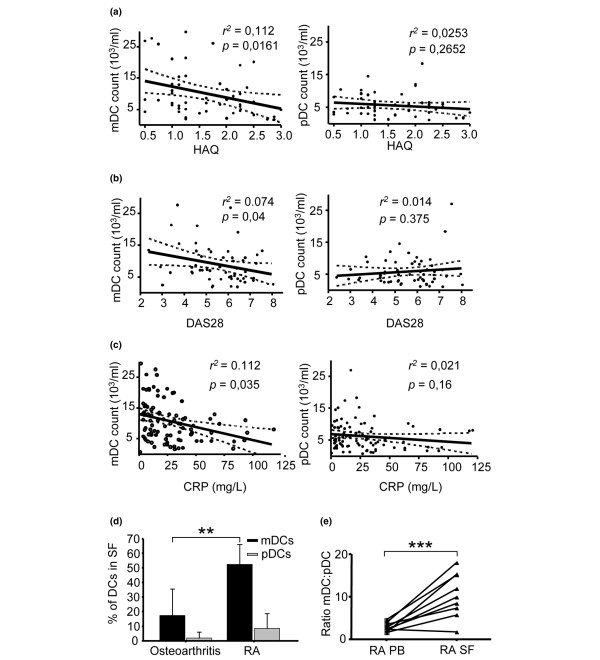
Correlation between circulating dendritic cell subsets and disease activity markers. Circulating plasmacytoid dendritic cell (pDC) and myeloid dendritic cell (mDC) counts (mean numbers/ml blood) from rheumatoid arthritis (RA) patients (n = 60) plotted against **(a) **Health Assessment Questionnaire (HAQ) score, **(b) **Disease Activity Score in 28 joints (DAS28), and **(c) **C-reactive protein (CRP) level. **(d) **The mDC level in synovial fluid (SF) of patients with active RA is significantly increased compared with that in osteoarthritis patients. Dendritic cell (DC) subsets were measured in the SF of patients with RA (n = 9) and in osteoarthritis patients (n = 11). The percentage of CD11c^+^CD123^-^HLADR^+ ^mDCs and CD11c^-^CD123^+^HLADR^+ ^pDCs in Lin^- ^cells (CD3^-^, CD14^-^, CD16^-^, CD56^-^, CD8^-^, CD19^-^) are shown (mean ± standard deviation). **P < 0.001, Mann–Whitney U test. **(e)** The mDC:pDC ratio in SF from RA subjects is significantly increased compared with the ratio in matched peripheral blood (PB) samples. Squares and triangles indicate individual matched samples (n = 9). The ratio is calculated from the percentage of mDCs and pDCs in Lin^- ^cells. ***P < 0.01, Mann–Whitney U test.

We then looked for a correlation between absolute counts of blood DCs and the clinical status or laboratory tests known to reflect disease activity (DAS28, Health Assessment Questionnaire score, and C-reactive protein level). In RA patients, mDC counts were inversely correlated with each of these markers (*P *< 0.05, *r*^2 ^= 0.07, *P *< 0.02, *r*^2 ^= 0.11 and *P *< 0.05, *r*^2 ^= 0.11, respectively, for DAS28, Health Assessment Questionnaire score and C-reactive protein level). We did not find any statistical correlation between the pDC counts and DAS28, Health Assessment Questionnaire score or C-reactive protein level (Figure 2a,b,c).

The levels of both DC subsets are therefore decreased in the blood of RA patients with active disease, but only mDCs correlate inversely with disease activity – suggesting that this mDC decrease could reflect a migration to inflamed tissues. Accordingly, we found a higher percentage of mDCs in synovial fluid from active RA patients compared with that from patients with osteoarthritis (percentage ± standard deviation: mDC = 52.5 ± 13.7% in the RA group vs. 17.4 ± 18.3% in the osteoarthritis control group; *P *= 0.0005). In contrast, the percentage of pDCs in synovial fluid was not different between the RA and the osteoarthritis groups (percentage ± standard deviation: pDC = 8.4 ± 10.9% in the RA group vs. 2 ± 3.9% in the osteoarthritis control group, *P *= 0.1119) (Figure 2d). The preferential migration of mDCs to inflamed joints was also suggested by the increase of the mDC:pDC ratio in synovial fluid compared with that found in peripheral blood (median, 3.8:1; *P *< 0.01, Wilcoxon matched-pairs test) (Figure 2e).

### Evolution of dendritic cell subset counts in infliximab-treated RA patients and correlation with the treatment response

Our initial results suggest that mDCs migrate from the blood to the inflamed synovial compartment. If this is the case, it seemed likely that effective therapy might block this migration and increase the blood mDC level.

**Figure 3:**
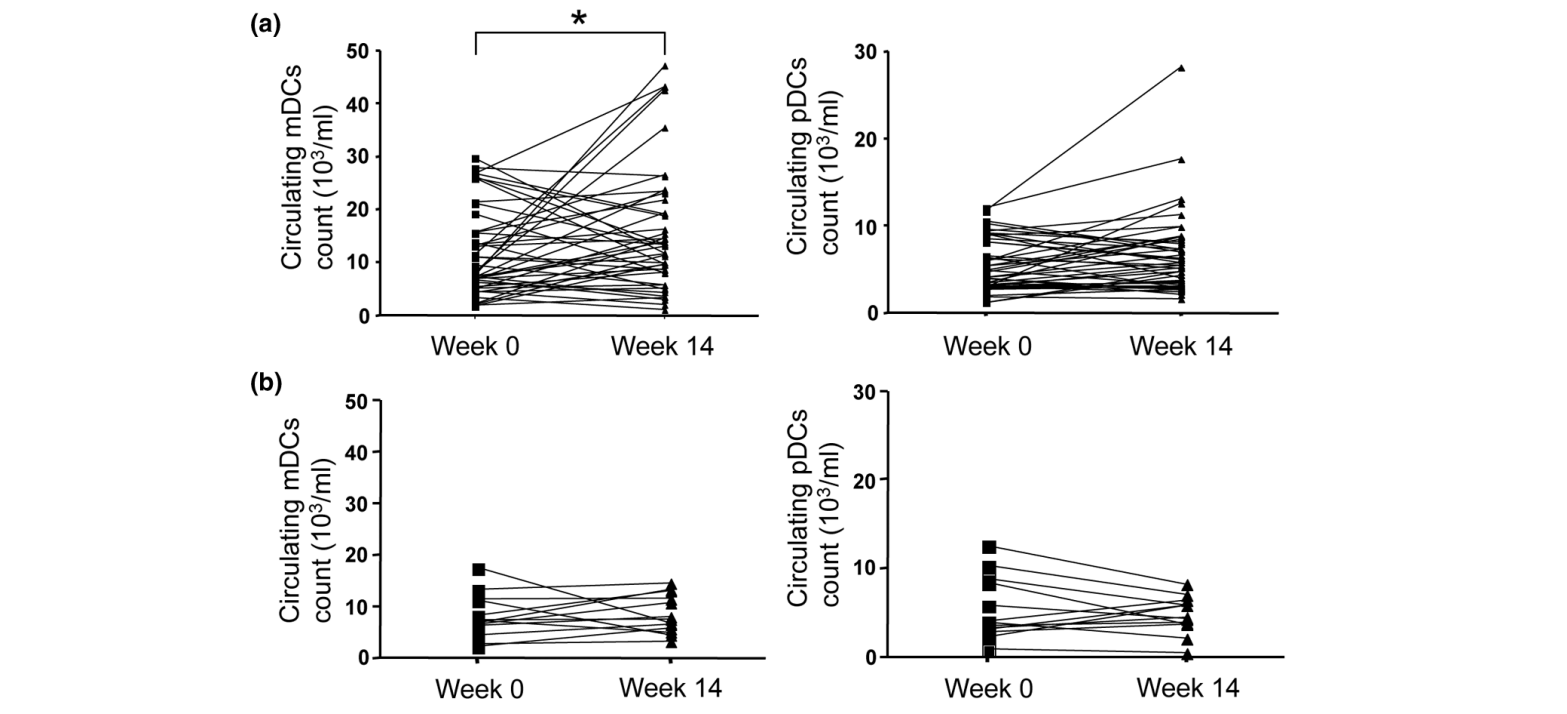
Evolution of circulating myeloid and plasmacytoid dendritic cell counts over 14-week treatment with infliximab. Evolution of circulating myeloid dendritic cell (mDC) and plasmacytoid dendritic cell (pDC) counts over a 14-week period of treatment with infliximab in **(a) **responder patients (n = 46) and **(b) **nonresponder patients (n = 13). Squares indicate matched samples. *P < 0.05, using Wilcoxon matched-pairs test.

Responders to the infliximab regimen (n = 46) were defined by a DAS28 decrease >1.2 after 14 weeks of infliximab therapy, whereas nonresponders (n = 13) were patients defined by a DAS28 variation <1.2 at week 14. Responders showed a substantial increase in their numbers of circulating mDCs (mean ± standard deviation = 11,915 ± 8,630 cells/ml at day 0 vs. 15,868 ± 11,467 cells/ml at week 14, *P *< 0.05 using Wilcoxon matched-pairs test) (Figure 3a), whereas the blood pDC level did not change significantly (5,632 ± 3,035 cells/ml at day 0 vs. 6,555 ± 4,656 cells/ml at week 14, *P *= 0.23) (Figure 3b). In contrast, nonresponders did not show statistically significant changes in mDC and pDC counts, and some patients even showing a decrease in both DC subsets during the course of treatment (mean ± standard deviation: mDCs = 7,991 ± 4,275 cells/ml at day 0 vs. 8,386 ± 3,689 cells/ml at week 14, *P *= 0.41; and pDCs= 5,542 ± 3,525 cells/ml at day 0 vs. 4,649 ± 2,032 cells/ml at week 14, *P *= 0.27) (Figure 3a,b). These data suggest the existence of a relationship between the fluctuations of the mDCs present in the blood and the variations of disease activity.

### Plasmacytoid dendritic cell number and blood IFNα levels correlate with anti-nuclear antibody positivity in infliximab-treated RA patients

The development of ANA is one of the most common side effects of TNFα-blocker therapies [25,26]. We therefore looked for a correlation between ANA appearance and DC count evolution in RA patients treated with infliximab.

The ANA levels were determined at the same time as the peripheral DC levels. After 14 weeks of treatment, we separated infliximab-treated patients into two groups: patients with positive ANA (n = 30) and patients with negative ANA (n = 16). The ANA level was considered positive when the serum dilution giving a positive signal in the indirect immunofluorescence on Hep-2 cells was above 1:250 and negative at the beginning of the treatment, or if the dilution increment reached at least three times the dilution observed at treatment onset. All of the data were obtained on day 0 of treatment onset and at week 14.

**Figure 4:**
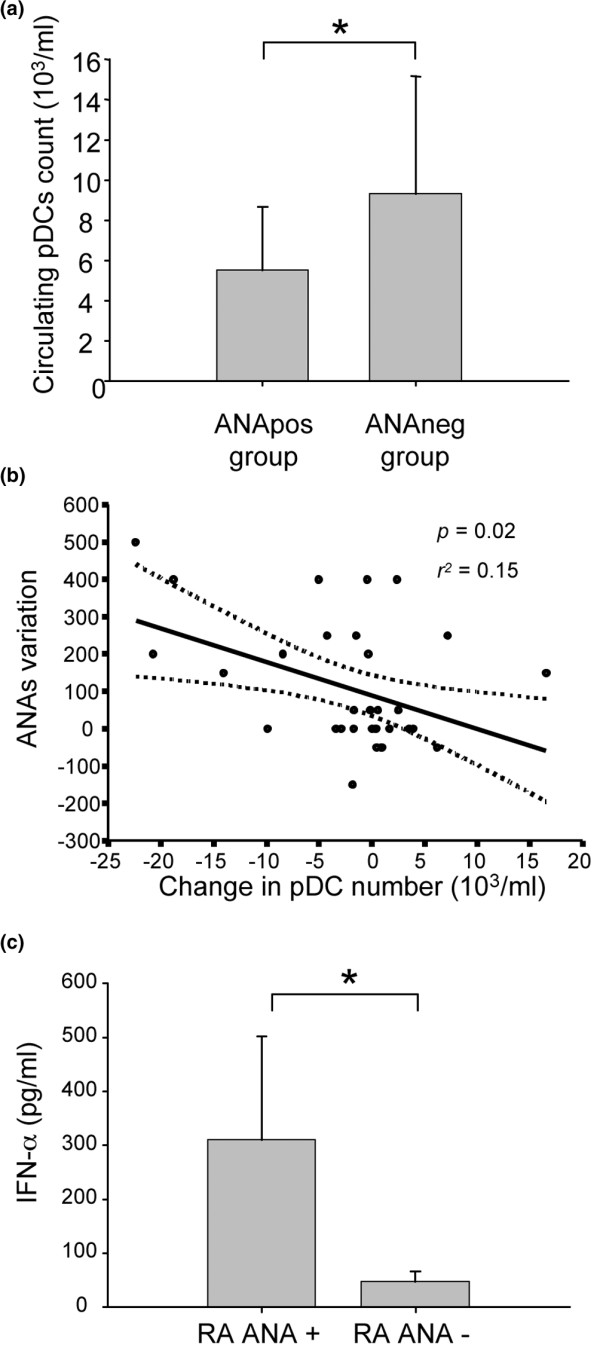
Plasmacytoid dendritic cell number, blood IFNα and anti-nuclear antibody positivity in infliximab-treated rheumatoid arthritis patients. **(a) **Circulating plasmacytoid dendritic cell (pDC) levels in rheumatoid arthritis (RA) patients after 14 weeks of infliximab therapy. pDC subsets were measured in the peripheral blood of patients, and two groups were individualized: patients with positive anti-nuclear antibody (ANA) (ANApos, n = 30) and patients with negative ANA (ANAneg, n = 16). Mean ± standard deviation shown. *P < 0.01, Mann–Whitney U test. **(b) **Correlation between ANA levels and pDC variations under infliximab therapy. ANA levels (1/dilution) and the pDC amount were measured on the same blood draw, before each infliximab infusion. **(c) **Detecting IFNα in serum of RA patients treated by infliximab and developing or not ANA. IFNα levels (pg/ml) were measured in peripheral blood of RA patients under infliximab therapy with ANA (n = 30) or without ANA (n = 16). *P < 0.001, Mann–Whitney U test.

At week 14, the pDC levels were statistically lower in the ANA-positive group when compared with the ANA-negative group (mean ± standard deviation: circulating pDCs = 5,509 ± 3,161 cells/ml vs. 9,324 ± 5,834 cells/ml, *P *< 0.01) (Figure 4a). Although no statistically significant difference was found in the mDC subset between the two groups (data not shown), the decrease of peripheral pDC counts correlated with the increase of ANA titers (*P *= 0.02, *r*^2 ^= 0.15) (Figure 4b).

Because IFNα and pDCs have been implicated in autoantibody production in SLE pathogenesis [3], we measured the IFNα level in the blood of both ANA-positive and ANA-negative RA patients treated by infliximab. We found that RA patients developing ANA were characterized by higher levels of IFNα (310 pg/ml vs. 47 pg/ml, *P *< 0.01), suggesting that infliximab influences pDC homeostasis and promotes the production of ANAs through the secretion of IFNα (Figure 4c).

### Anti-TNFα antibody infliximab keeps plasmacytoid dendritic cells in an IFNα secreting state

**Figure 5:**
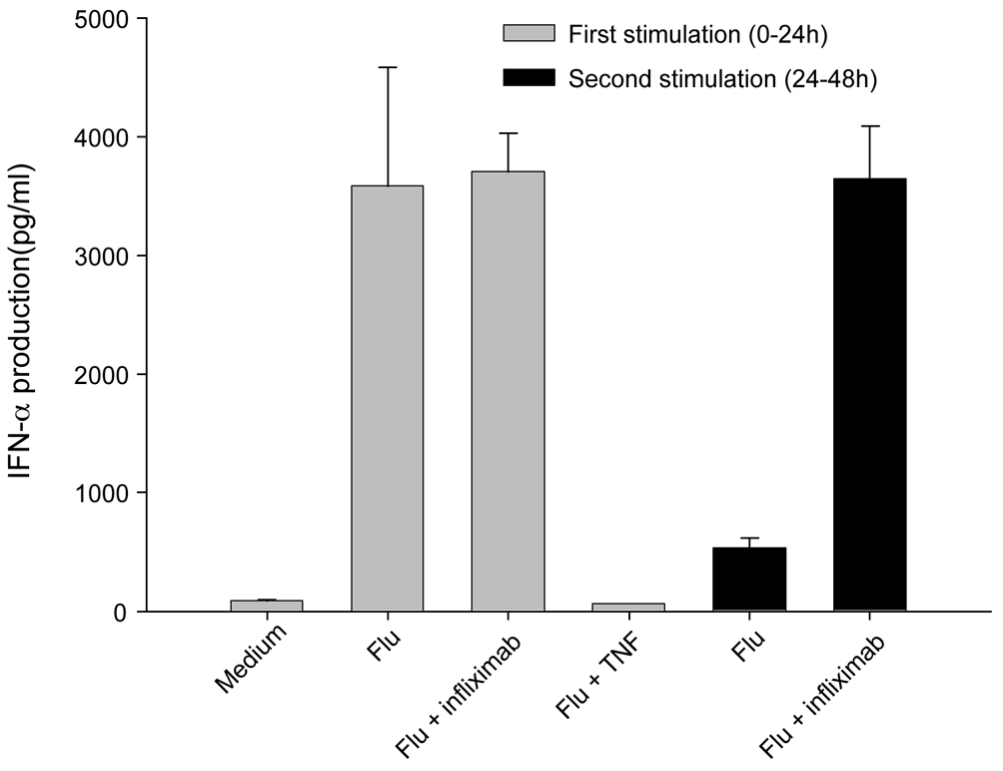
Infliximab maintains plasmacytoid dendritic cells exposed to influenza virus in an IFNα secreting state. Peripheral blood mononuclear cells from control donors were exposed to influenza virus alone (Flu) or to influenza virus in the presence of infliximab. After 24 hours of incubation, the supernatant was collected and IFNα levels were measured by ELISA. The cell pellets were then washed, resuspended in fresh medium, and exposed for an additional 24 hours to influenza virus. Supernatants were analyzed by ELISA. Data are expressed as the mean ± standard error of the mean of three independent experiments.

The presence of higher amounts of IFNα in RA ANA-positive patients prompted us to analyze the effects of infliximab on pDCs' ability to secrete IFNα in vitro. PBMCs from control donors were exposed to influenza virus alone or in the presence of infliximab. Influenza virus was used as a well-known strong pDC-IFNα inducer. We did not find any increase in cellular apoptosis of the cells in any of the conditions tested (data not shown). In both conditions (virus alone or virus + infliximab), we detected high levels of IFNα in the supernatant collected after 24 hours culture, without any differences between the two conditions (Figure 5). Repeat exposure of PMBCs to influenza virus, however, was able to induce large IFNα production only in the presence of infliximab. Furthermore, PBMCs pretreated with TNFα were unable to secrete significant amounts of IFNα. Although these studies were performed with PBMCs, it is probable that pDCs were the major source of IFNα given that they are the major IFNα-producing cells in peripheral blood. These data strongly suggest that infliximab maintains pDCs in an IFNα secreting state by quenching TNFα.

### Infliximab increases plasma cell generation and promotes *in vitro *anti-nuclear antibody secretion

**Figure 6:**
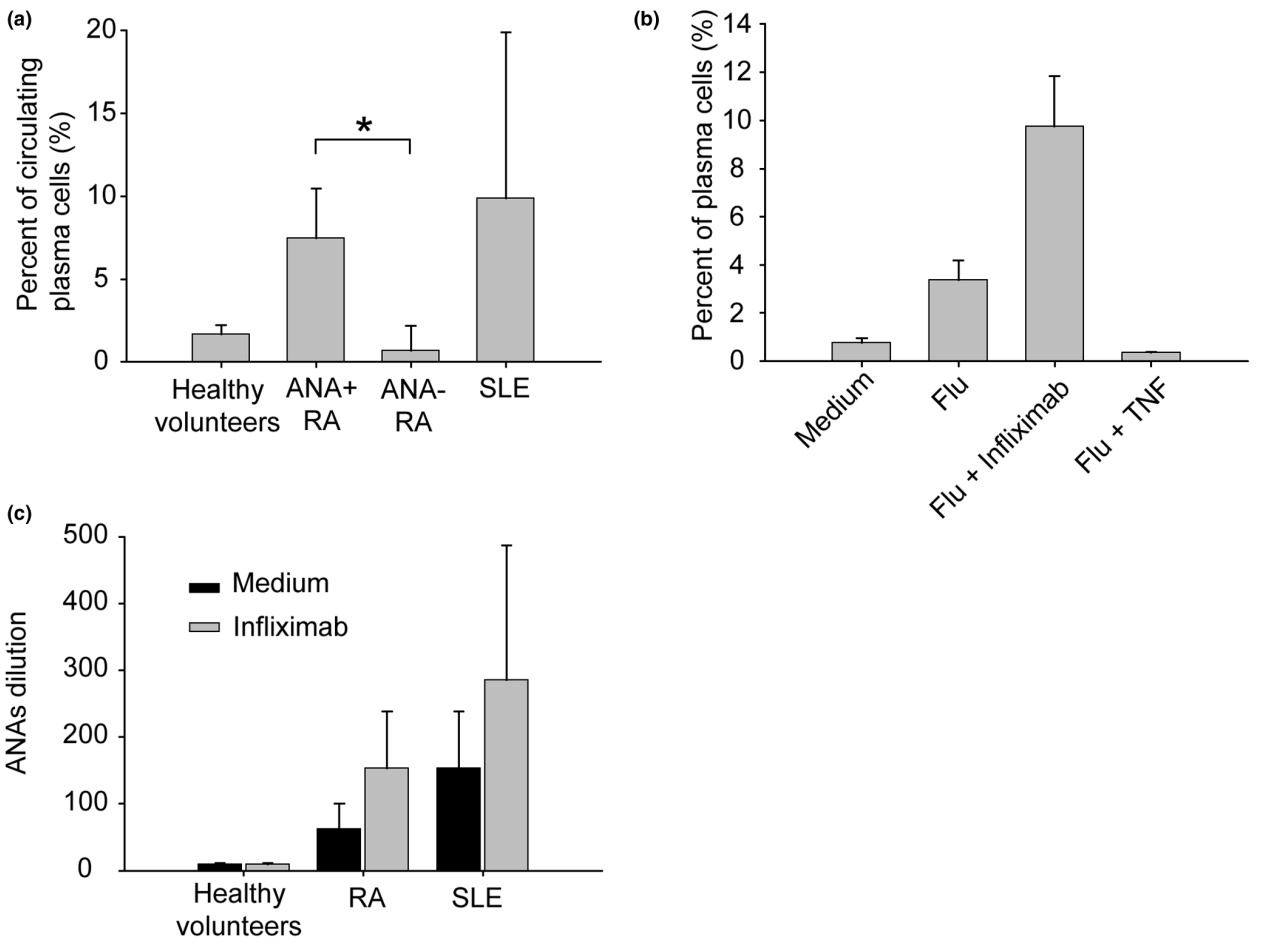
Infliximab enhances plasma cell differentiation. **(a) **Circulating plasma cell levels in rheumatoid arthritis (RA) patients treated by infliximab with anti-nuclear antibody (ANA) (n = 10) or without ANA production (n = 10), in patients with active systemic lupus erythematosus (SLE) (n = 10), and in healthy volunteers (n = 10). Plasma cell levels were measured in peripheral blood. The mean number/milliliter of CD38^+^CD19^+^CD20^- ^is shown (mean ± standard deviation). *P < 0.001, Mann–Whitney U test. **(b) **Peripheral blood mononuclear cells (PBMCs) from healthy donors were cultured out in the presence of influenza virus (Flu) with or without infliximab or TNFα. After 10 days, we analyzed by flow cytometry the proportion of CD19^+^CD20^-^CD38^high+ ^plasma cells. Data expressed as the mean ± standard error of the mean of three independent experiments. **(c) **PBMCs from healthy donors, from RA patients treated by infliximab and developing ANAs, and from SLE patients were cultured in the presence of influenza virus with or without the TNFα blocker, infliximab. After 15 days, ANA titers were measured in the supernatants. Data expressed as mean ± standard error of the mean of three independent experiments.

Jego and colleagues showed that pDCs exposed to viral infection were able to activate the B-lymphocyte compartment and to promote the generation of plasma cells and/or plasmablasts in an IFNα-dependent and IL-6-dependent fashion [4]. To delineate the consequences of the sustained IFNα secretion induced by infliximab, we compared the proportion of circulating CD19^+^CD20^-^CD38^+ ^plasma cells in RA ANA-positive patients (n = 10) and RA ANA-negative patients (n = 10). RA ANA-positive patients exhibited a significant increase (*P *< 0.001) of the proportion of circulating plasma cells compared with RA ANA-negative patients (Figure 6a). The percentage of plasma cells in RA ANA-positive patients was similar to that observed in SLE patients.

We then tested, *in vitro*, whether infliximab effects plasma cell generation from B lymphocytes. PBMCs were cultured with influenza virus with or without infliximab. After 15 days, we measured the proportion of CD19^+^CD20^-^CD38^high+ ^plasma cells. PBMCs cultured with influenza virus + infliximab were characterized by a higher proportion of plasma cells. Interestingly, concomitant addition of TNFα with virus stimulation inhibited plasma cell generation (Figure 6b). To analyze the *in vitro *effects of infliximab on ANA secretion, we repeated the experiment with PBMCs from RA ANA-positive patients, SLE patients and healthy control individuals. These PBMCs were cultured for 15 days in the presence of influenza virus with or without infliximab. After 15 days the secretion of ANA was found only in supernatants from cells from RA ANA-positive patients or SLE patients, and was further increased in the presence of infliximab (Figure 6c).

Taken together, those results suggest that infliximab promotes pDCs in an IFNα secreting state and allows for the differentiation of B lymphocytes into ANA-secreting plasma cells.

## Discussion

DCs are thought to play a key role in driving the immunopathogenic response underlying chronic inflammatory arthritis. Various studies [9,27-29] have shown that both mDCs and pDCs accumulate in synovial tissue and synovial fluid of RA patients. The evolution of circulating peripheral blood DC counts under TNFα blocker therapy has never been studied, however, but it may provide important information on the implication of both subsets in RA pathogenesis.

In the present study we show that RA patients are characterized by a significant decrease in circulating mDCs and pDCs, consistent with previous results from Jongbloed and colleagues [11]. We, however, found that only mDC counts correlated inversely with RA activity as assessed by the Health Assessment Questionnaire score, DAS28, and C-reactive protein level, and that the percentage of mDC was increased in the inflamed synovial tissue. Moreover, in the presence of effective infliximab therapy, circulating mDC counts increased to reach levels observed in healthy volunteers. Our results suggest that, among DCs, mDCs have a prominent role in clinical disease manifestations in RA patients since their circulating numbers correlate directly with disease activity, and treatment with infliximab corrects mDC count abnormalities in infliximab-responsive patients.

The lack of correlation between pDC counts and RA clinical evolution was unexpected because pDCs are known to play a central role in various inflammatory diseases, including psoriasis [30], Sjogren's syndrome [31] and SLE [3]. It is probable that other unknown parameters may alter pDC homeostasis in RA patients. Psoriasis [32-34] and SLE (or more frequently the appearance of ANA) [12-16] have been described as an adverse effect of TNFα-blocker therapy. In both diseases, pDCs are implicated in pathogenesis through their ability to produce high amounts of IFNα [3,30]. In the case of SLE, this occurs through uptake of the immune complex on the pDC cell surface and the subsequent internalization and delivery of the self-DNA or self-RNA within the complex to intracellular TLR9 or TLR7, respectively [35-37]. In the case of psoriasis, the endogenous antimicrobial peptide LL37 forms a complex with self-DNA that is delivered to and retained within early endocytic compartments of pDCs to trigger TLR9 and to induce IFNα production [38]. Interestingly, a recent study has reported an increased IFNα expression and more severe psoriatic skin lesions in patients treated with TNF blockers [34], implicating IFNα in the pathogenesis of psoriasis.

As previously proposed by Palucka and colleagues in systemic-onset juvenile idiopathic arthritis [39], we thought pDCs may be preferentially involved in the ANA response frequently found in RA patients – which increases under treatment. Indeed, we found a direct correlation between ANA levels and decreased pDC variation. Furthermore, serum IFNα was significantly increased in patients developing ANAs. Several studies [39-41] evaluating IFNα production in autoimmune diseases have measured IFNα gene expression and IFN-inducible gene expression instead of measuring serum IFNα protein levels because of the limited sensitivity of the ELISA assay. In our study, however, the serum level of IFNα induced by influenza was high enough to be detected at the protein level, allowing the same ELISA assay to be used for both *in vivo *and *in vitro *measurement of IFNα. Our results suggest that migration of pDCs – which are known to enter lymph nodes when they produce IFNα [42] – occurs, leading to their decreased numbers at the periphery in the ANA-positive group. Moreover, this IFNα secretion from pDCs has been previously described to induce plasma cell differentiation and, therefore, autoantibody production [4]. Accordingly, *in vivo*, we found increased plasma cell generation in RA patients developing ANAs during infliximab therapy.

IFNα-secreting pDCs have been described as being immature or precursor DCs [43]. TNFα is known to differentiate immature DCs into a more mature stage [44] and to inhibit IFNα induced by viruses [45]. TNFα-mediated maturation of pDCs could block the IFNα-producing ability of pDCs. Conversely, pDCs stimulated by viruses secrete high amount of IFNα and TNFα that could act in an autocrine loop to control IFNα secretion through pDC maturation.

We confirm that TNFα blocks the ability of pDCs to secrete IFNα upon viral stimulation, and that the TNFα antagonist, infliximab, keeps pDCs in an IFNα secreting state. This result and our *in vivo *data described above are consistent with a previous report showing that, *in vitro*, TNFα blockers inhibit virus-induced maturation of pDCs and increase IFNα secretion [39]. The authors suggested that this inhibition may explain the increase of ANA production in patients treated with TNFα blockers. We confirmed their findings by showing, *in vitro *and *in vivo*, the ability of infliximab to increase IFNα secretion, plasma cell differentiation and ANA generation. de Rycke and colleagues, however, have previously described differences in ANA induction between infliximab and etanercept in patients suffering from spondylarthropathy [13]. It will therefore be important to determine in future work whether other TNF blockers (adalimumab and etanercept) have the same ability as infliximab to maintain pDCs in an IFNα secreting state.

## Conclusions

Although both subtypes of circulating DCs are reduced in active RA patients' peripheral blood, only mDC levels correlated with disease activity, suggesting a possible link to RA pathogenesis. The exact role of pDCs in RA remains unclear, but these cells seem likely to play an important role in lupus-like complications of infliximab therapy as they do in lupus.

We confirmed that infliximab acts on the regulation of IFNα system *in vivo *and *in vitro*, by enhancing plasma cell differentiation, which is ultimately responsible for autoantibody secretion. Our results emphasize the balance between IFNα and TNFα in RA, and provide mechanistic insights into the possible roles of DC subsets in mediating the shift in autoimmune disease manifestations by therapeutics that inhibit TNFα. These findings may also be relevant in other autoimmune diseases where the role of IFNα and TNFα has been suggested, such as psoriasis [30,46].

## Abbreviations

ANA: anti-nuclear antibody; DAS28: Disease Activity Score in 28 joints; DC: dendritic cell; ELISA: enzyme-linked immunosorbent assay; EULAR: European league against rheumatism; FCS: fetal calf serum; IFN: interferon; IL: interleukin; mDC: myeloid dendritic cell; PBMC: peripheral blood mononuclear cell; PBS: phosphate-buffered saline; pDC: plasmacytoid dendritic cell; RA: rheumatoid arthritis; SLE: systemic lupus erythematosus; TNF: tumor necrosis factor.

## Competing interests

The authors declare that they have no competing interests.

## Authors' contributions

CR, TS, J-FM and PB designed the study. CR and CD collected clinical patient data. CR, TS and PB performed all experiments and analyzed the data. CR and PB drafted the manuscript. JD followed up the patients. All authors read and approved the final document.
